# The Retrospective Address Delivered at the Ninth Anniversary Meeting of the Provincial Medical and Surgical Association

**Published:** 1842-04

**Authors:** 


Art. VIII.—*The Retrospective Address delivered at the Ninth Anniversary Meeting of the Provincial Medical and Surgical Association, held at York, August 4th and 5th,* 1841. By R. J. N. Streeten, m.d., Physician to the Worcester Dispensary.—*Worcester,* 1841. 8vo, pp. 104.

This excellent address is well worth the attention of the profession as containing, in a very condensed form, all the improvements made in the medical sciences during the preceding twelve months. It shows, at once, extensive reading and much good judgment on the part of its author. It is unsusceptible of analysis, and our limited space forbids extract.

